# Radical Addition to Iminium Ions and Cationic Heterocycles

**DOI:** 10.3390/molecules191016190

**Published:** 2014-10-10

**Authors:** Johannes Tauber, Dennis Imbri, Till Opatz

**Affiliations:** Institute of Organic Chemistry, Johannes Gutenberg-University Mainz, Duesbergweg 10–14, 55128 Mainz, Germany

**Keywords:** radicals, iminium salts, heterocycles, addition reactions, rearrangements, Minisci reaction, Porta reaction, photoredox catalysis, transition metal catalysis, late-stage functionalization

## Abstract

Carbon-centered radicals represent highly useful reactive intermediates in organic synthesis. Their nucleophilic character is reflected by fast additions to electron deficient C=X double bonds as present in iminium ions or cationic heterocycles. This review covers diverse reactions of preformed or *in situ*-generated cationic substrates with various types of C-radicals, including alkyl, alkoxyalkyl, trifluoromethyl, aryl, acyl, carbamoyl, and alkoxycarbonyl species. Despite its high reactivity, the strong interaction of the radical’s SOMO with the LUMO of the cation frequently results in a high regioselectivity. Intra- and intermolecular processes such as the Minisci reaction, the Porta reaction, and the Knabe rearrangement will be discussed along with transition metal and photoredox catalysis or electrochemical methods to generate the odd-electron species.

## 1. Introduction

Radicals, representing odd-electron species, possess the peculiarity of combining nucleophilicity and electrophilicity at the same reaction center since their HOMO and LUMO (highest occupied/lowest unoccupied molecular orbital) are located in one common molecular orbital, the SOMO (singly occupied molecular orbital). As a consequence, radical reactions are less influenced by Coulombic attraction than polar reactions but are rather governed by orbital interactions to a large extent. Thus, there is an analogy between radicals and “soft” electrophiles or nucleophiles for which orbital interactions are also primarily responsible for controlling their chemical reactivity [1].

**Figure 1:**
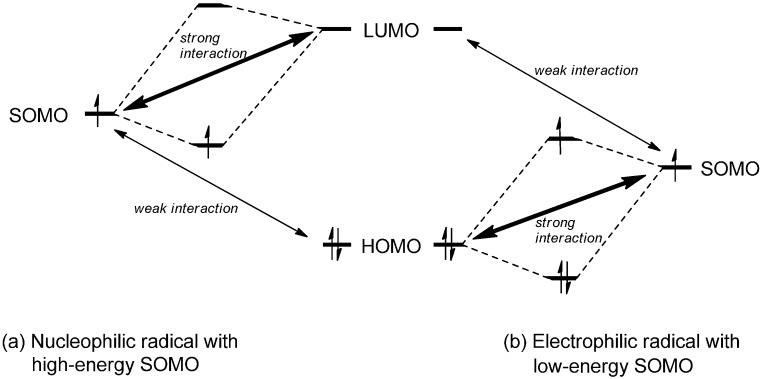
Interactions of discrete SOMO energy levels with the HOMO/LUMO of a molecule and frontier orbital interaction for a nucleophilic and an electrophilic radical.

Being typically located in energy between the HOMO and the LUMO of the reaction partner, the overlap of the radical’s SOMO with both frontier orbitals will be energetically favorable. Depending on the SOMO energy, either the SOMO-HOMO or the SOMO-LUMO interaction may be dominant as the result of a smaller energy difference. In the former case, the radical can be classified as electrophilic while nucleophilic properties can be ascribed to it in the latter case (Figure 1) [1].

Carbenium ions and carbon-centered radicals share the same tendency of increased stability from primary to tertiary carbons which can be interpreted in terms of increasing hyperconjugative stabilization (*vide infra*). Usually, carbon-centered radicals are viewed as nucleophilic species with their nucleophilicity increasing with the degree of alkyl substitution or the presence of adjacent lone pairs as in the case of alkoxymethyl or hydroxymethyl but this of course strongly depends on the nature of the particular interaction partner. Thus, even radicals with an estimated electrophilic character are actually capable of reacting as nucleophiles if a reactant with a low lying LUMO is present. This behavior is an important aspect of the characteristic reactivity of radicals, making them a powerful, yet sometimes less predictable tool for organic synthesis.

## 2. Addition of Carbon-Centered Radicals to Cationic Heterocycles

### 2.1. Theoretical Background, SOMO-HOMO/LUMO Interaction

Frontier orbitals provide valuable information to explain reactivity and selectivity of the addition of carbon-centered radicals to electron deficient heteroaromatic substrates. Alkyl radicals have been found to preferentially attack pyridinium cations, a reaction first described by Minisci in 1971 as a homolytic alkylation of heteroaromatic bases [2], and are consequently classified as nucleophilic. This is also reflected by the regioselectivity of the reaction exclusively taking place in the 2- and 4-position of the pyridine core.

**Figure 2:**
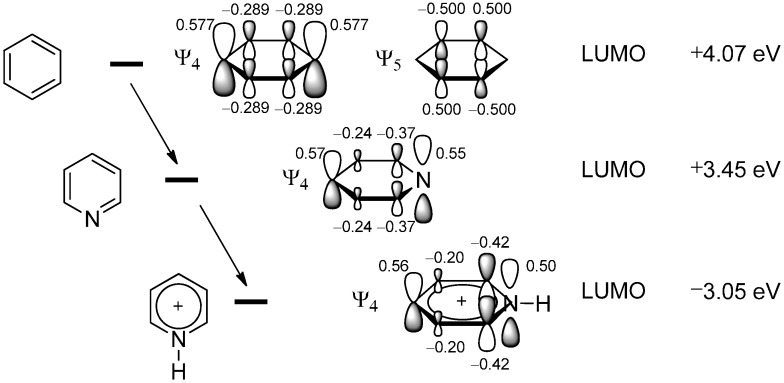
Coherence between reactivity towards nucleophilic attack and the lowering of the LUMO’s energy (values calculated with Gaussian09 D01 at the HF/6-31G* level of theory [4] as reported in the literature for the pyridinium ion [5]. The MO-coefficients are based on Hückel theory).

Frontier molecular orbital theory not only correctly predicts the reactive positions of the pyridinium ion as those with the largest coefficients in the LUMO but also explains the much higher reactivity of the cationic form with its significantly lowered LUMO energy compared to neutral pyridine which itself is more reactive than benzene (Figure 2) [1,3].

**Figure 3:**
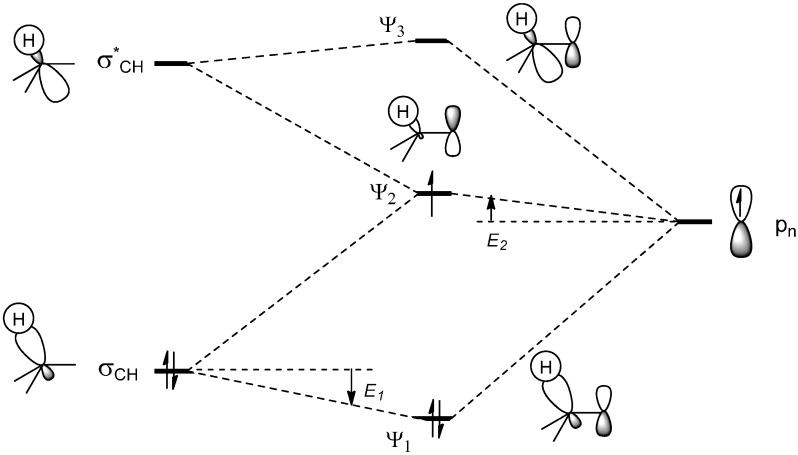
Interaction of the orbitals of a σ_(C–H)_-bond with a singly-occupied non-bonding 2p-orbital, comparable to the stabilization by hyperconjugation in carbenium ions.

The absolute reactivity in terms of the reaction rate of an alkyl radical with a heteroarene is affected by various factors including ionization potential, electron affinity, bond localization energy, and superdelocalizability [1]. Steric factors may also play an important role. Due to hyperconjugation with the adjacent σ_(C–H)_-orbitals, tertiary alkyl radicals have a higher SOMO energy than their secondary or primary counterparts and show an increased nucleophilic behavior (Figure 3).

**Figure 4:**
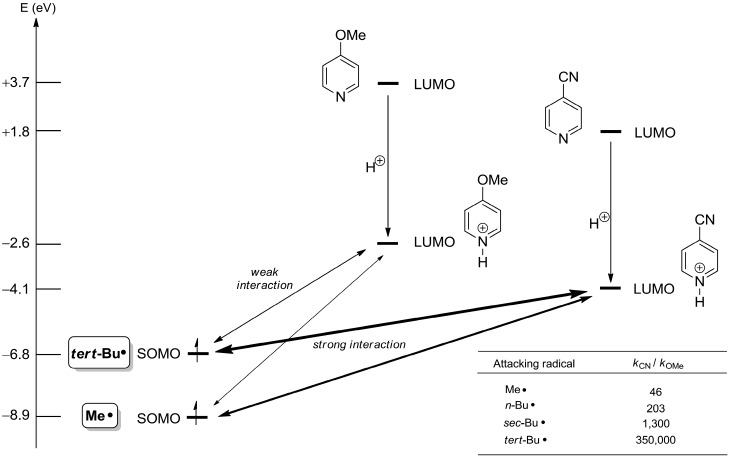
Interactions of frontier orbitals of alkyl radicals with pyridinium cations (values calculated with Gaussian09 D01 at the HF/6-31G* level of theory [4], SOMO energy = −IP) [6].

This not only increases their net absolute reactivity but also their selectivity in competition experiments. A prominent example is the *ortho*-alkylation of the 4-cyanopyridinium and the 4-methoxypyridinium cation by primary, secondary and tertiary alkyl radicals in which the relative kinetic preference (k_CN_/k_OMe_) for the more electron-deficient 4-cyano derivative increases from 46 for the methyl radical over 1,300 for the *sec*-butyl radical to 350,000 for the *tert*-butyl radical (Figure 4) [1].

This nicely illustrates the role of the SOMO/LUMO energy gap for this process: the stronger the electron withdrawing effect of the 4-substituent, the lower will the LUMO’s energy value be for the pyridinium cation and the smaller the energy gap between the reacting frontier orbitals, the more willingly the reaction takes place. The following sections will provide further examples for this general principle of chemical reactivity.

### 2.2. The Minisci Reaction

**Scheme 1:**
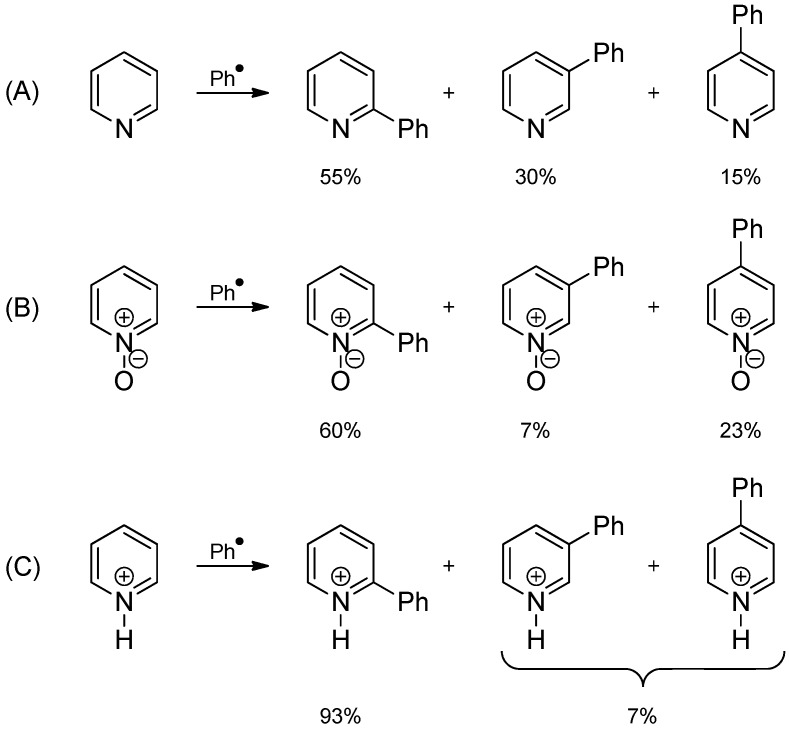
Radical phenylation of pyridine (**A**); pyridine-*N*-oxide (**B**); and protonated pyridine (**C**).

Homolytic aromatic substitution already played a role in an early era of organic chemistry [7]. In the early 1890s, the addition of aryl radicals to heteroaromatic systems had been first described, e.g., the phenylation of pyridine. The analogous radical phenylation of benzene to form biphenyl, the famous Gomberg-Bachmann reaction, was not reported until 1924 [8]. An important issue of these reactions has always been the lack of regioselectivity that usually leads to the formation of regioisomeric mixtures which may be difficult to separate. In the case of heterocyclic substrates, the regioselectivity could slightly be improved by using pyridine-*N*-oxide (Scheme 1) [9]. Lynch, Chang, and Dou discovered the significant effect of the acidity of the reaction medium, *i.e.*, that the degree of N-protonation of the heteroaromatic substrate, does not only alter the regioselectivity but also enhances the reaction rate compared to the neutral bases for pyridine, *N*-methylimidazole, thiazole, quinoline, isoquinoline, benzothiazole, pyrazine, pyrimidine, quinoxaline, and *N*-methylbenzimidazole [9,10,11,12,13,14,15].

**Scheme 2:**
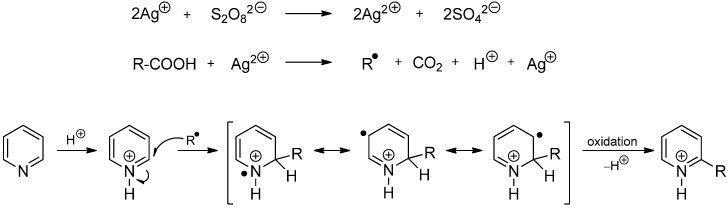
Nucleophilic radical addition to a protonated pyridinium ion (Minisci reaction).

In 1971, Minisci established a new method for the generation of alkyl radicals by silver-catalyzed oxidative decarboxylation of carboxylic acids with peroxydisulfate and demonstrated the positive effect of acidic conditions on the homolytic alkylation of heteroarenes (Scheme 2) [2]. Since then, the reaction has been expanded tremendously and has evolved into a very effective method for the functionalization of electron-deficient heteroaromatics. For this reason, homolytic substitutions of protonated heteroarenes are often called “Minisci reactions”. The alkyl radical adds to the cationic substrate to afford an aminyl radical cation which is subsequently oxidized to the corresponding alkylated compound under the reaction conditions.

**Scheme 3:**
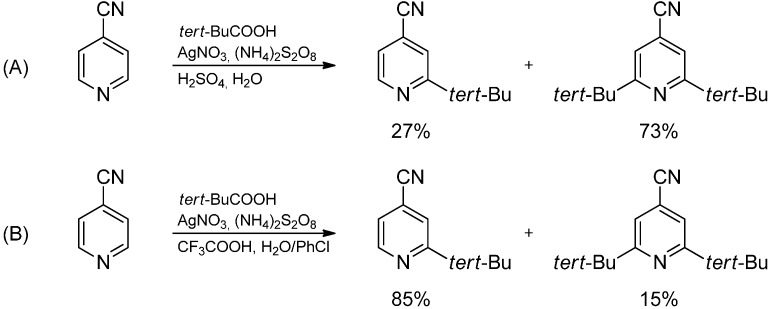
Alkylation of 4-cyanopyridine in water (**A**) and a two-phase system (**B**).

The reaction resembles the Friedel-Crafts-alkylation but the reactivity and selectivity is inverted as more electron-deficient heteroarenes react faster. The radical alkylation of the heteroaromatic base is accompanied by a slight increase of the LUMO energy due to the electron donating effect of the alkyl substituent. Nevertheless, polyalkylation is observed as the energy difference does not suffice to clearly disfavor consecutive reactions. Monoalkylation is however attainable by working in a two-phase system, in which the alkylated reaction product—the more lipophilic compound—is extracted from the aqueous phase (Scheme 3) [16].

**Scheme 4:**
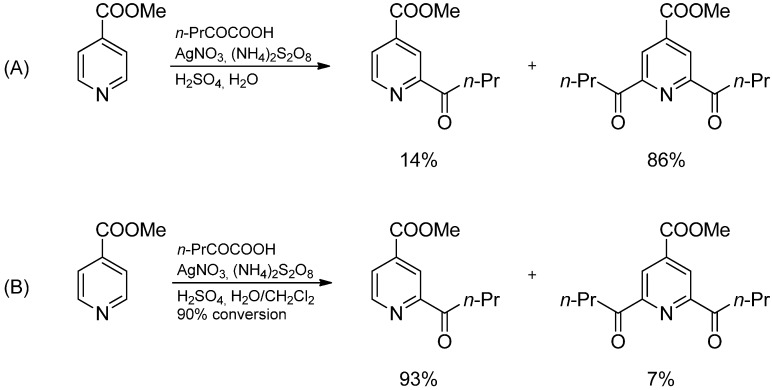
Acylation of 4-acetylpyridine in water (**A**) and a two-phase system (**B**).

The Minisci reaction can also yield acylated products if the aromatic compound is attacked by an acyl radical. These acylated compounds also appear as side products when carboxylic acids are used as the radical source as the homolytic cleavage of the C–OH bond may compete with oxidative decarboxylation. In contrast to the Friedel-Crafts-acylation, the first acylation increases the electron-deficiency of the hetarene significantly and will speed up any following radical addition/substitution by lowering the LUMO energy. Therefore, the isolation of the monoacylated product was cumbersome. Again, this problem could be solved by working in a two-phase system since the acylation decreases the basicity of the hetarene and increases the fraction of unprotonated compound which is extracted by the organic solvent, while the protonated starting material is retained in the aqueous phase where the radicals are being generated (Scheme 4) [16].

**Scheme 5:**
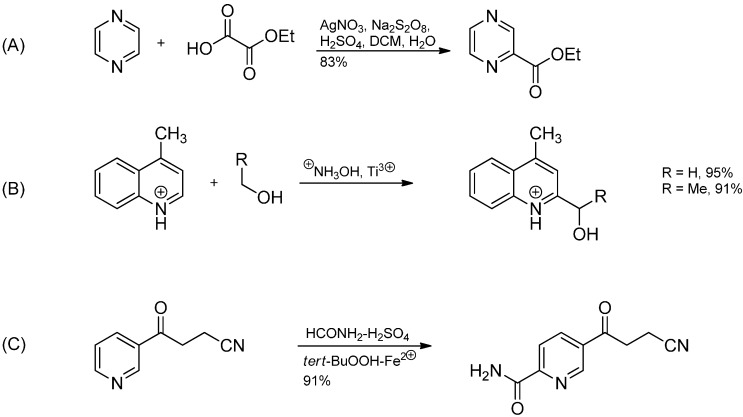
(**A**) Hydroxycarbonylation of pyrazine; (**B**) Hydroxyalkylation of 4-methylquinoline; (**C**) Carbamoylation of 4-oxo-4-(3-pyridyl)butyronitrile.

Besides the mentioned alkylations and acylations, other functionalizations of heteroaromatics are also known. If alcohols are used as the source of the carbon-centered radicals, hydroxyalkylated heterocyles can be obtained. The adjacent OH group with its high–lying n-electrons leads to a large increase in the radical’s SOMO energy, making it a particularly strong nucleophile. In the example depicted in Scheme 5B, an aminium radical generated by one-electron reduction of protonated hydroxylamine with Ti^3+^ and subsequent scission of the N–O bond abstracts an α-H-atom from the alcohol.

The reaction of pyrazine with oxalic acid monoesters under oxidative conditions leads to hydroxycarbonylation while carbamoylation can be achieved by using formamide in combination with a variant of Fenton’s reagent intermediately generating *tert*-butoxyl radicals.

As the oxidative decarboxylation of aromatic carboxylic acids is not feasible, the utilization of aryl radicals was restricted. However, further developments enabled the utilization of arylboronic acids as the radical source under similar oxidative conditions (AgNO_3_/peroxydisulfate) [17]. Both electron-poor and electron-rich arylboronic acids can be employed and alkoxy, aryloxy as well as halide substituents are tolerated. An exception are *ortho*-substituents on the arylboronic acid which hinder the reaction immensely and only electron-donor groups allow the reaction to occur in low yield.

**Scheme 6:**
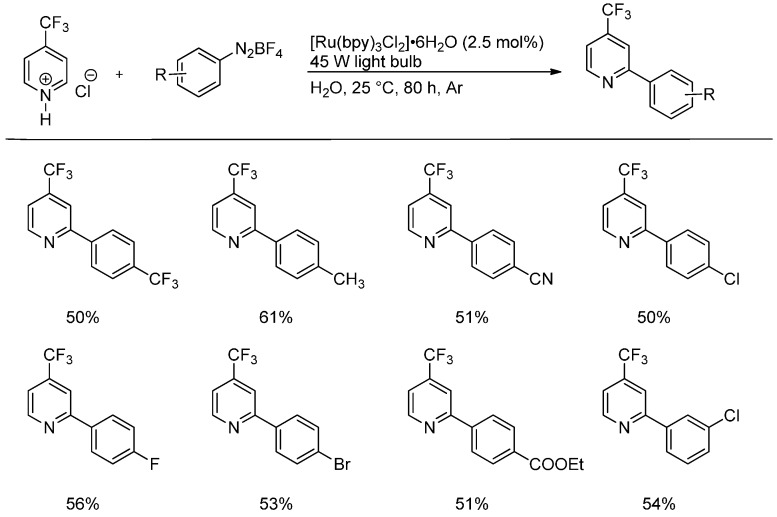
Arylation of nitrogen heterocycles with aryldiazonium salts by photoredox catalysis.

Recently, the arylation of pyridines has been reported by Xue and coworkers [18]. They generated aryl radicals from aryldiazonium salts by visible light-promoted photoredox catalysis using the popular Ru(bpy)_3_^2+^-system (Scheme 6). Many functional groups such as CN, CO_2_Et, F, Cl, Br, and CF_3_ are tolerated. Other heterocyclic substrates reacted under the same conditions (*vide infra*, Scheme 10).

**Scheme 7:**
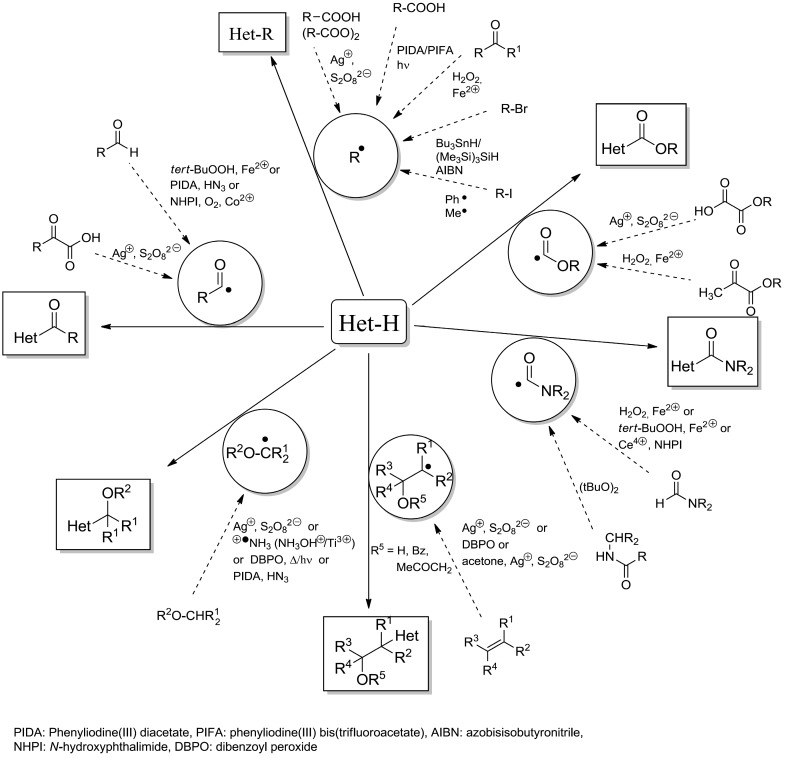
Versatility of the Minisci-reaction and variety of radical generation.

The versatility of the Minisci reaction is shown in Scheme 7 and a broad spectrum of heterocyclic bases (pyridine, quinoline, isoquinoline, triazole, phenazine, quinoxaline, benzothiazole, purine *etc.*) can be employed. Further details such as mechanistic investigations, rate constants for the radical addition, *etc.* can be found in specific reviews on the Minisci reaction [9,16,19,20,21,22,23,24].

Below, selected examples for the wide range of heteroarenes and the observed regioselectivity of the Minisci reaction will be presented. Recently, Baran, Blackmond, and O’Hara have devised several rules with respect to the effect of substituents which allow prediction and adjustment of the regioselectivity in different classes of nitrogen heterocycles [25]. Their observation of the dependence of the regioselectivity for the attack of CF_3_ radicals to acceptor substituted pyridines on the solvent may be interpreted in terms of H-bonding involving the pyridine nitrogen, enhancing the reactivity of the adjacent positions.

**Scheme 8:**
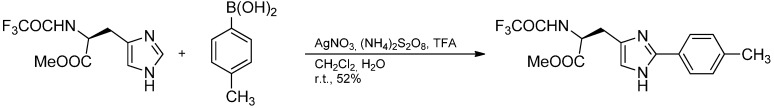
2-Arylation of histidine with an arylboronic acid in a Minisci-reaction.

Imidazole and 1-alkylimidazoles are usually attacked in the 2-position [26,27]. An example is the arylation of histidine with aryl radicals generated by oxidation of arylboronic acids (Scheme 8) [28]. Likewise, pyrazole is attacked at C3 [26].

**Scheme 9:**
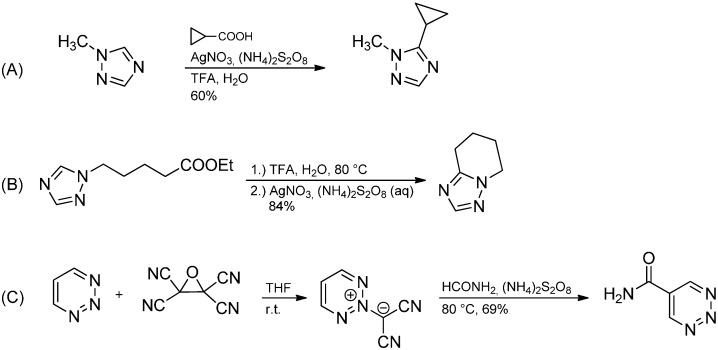
(**A**) Alkylation of *N*-alkyl 1,2,4-triazoles; (**B**) Formation of a bicyclic ring system with a 1,2,4-triazole moiety; (**C**) Carbamoylation of 1,2,3-triazine activated as a dicyanomethine ylide.

1-Alkyl-1,2,4-triazoles are alkylated in 5-position and 1,2,4-triazoles bearing a side chain with a carboxylic acid afford bicyclic ring systems in high yields (Scheme 9) [29,30]. 1,2,3-Triazine, which is unstable under the usual acidic conditions, has been activated by formal addition of dicyanocarbene instead of protonation. Conversion with formamide yields the carbamoylated 1,2,3-triazine regioselectively in the 5-position [30,31].

**Scheme 10:**
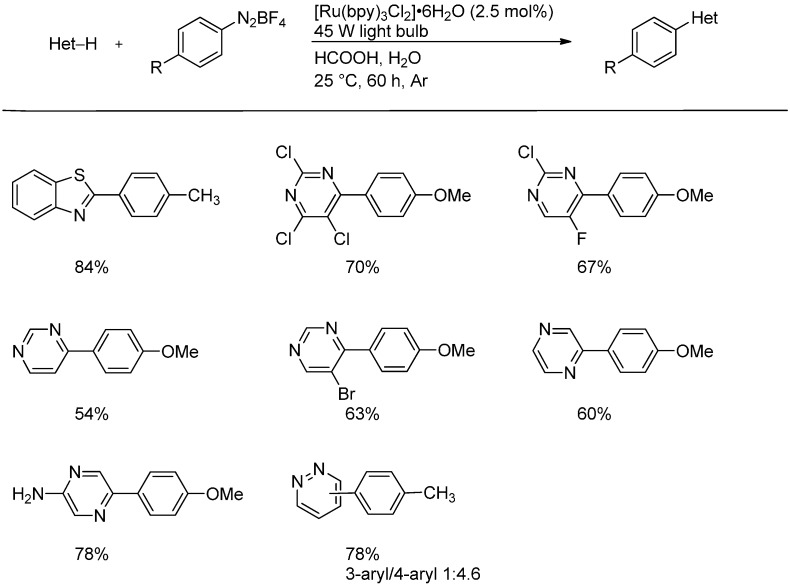
Arylation of heteroarenes with aryldiazonium salts by photoredox catalysis.

In addition to the previously presented method of radical generation via photoredox catalysis from aryldiazonium salts, Xue *et al.* demonstrated the versatility of their reaction on various heteroaromatic scaffolds which further underlines the general applicability of Minisci-type reactions (Scheme 10). It is noteworthy that pyrimidines show a general regioselectivity towards the 4-position while benzothiazoles preferably react in the 2-position. In contrast to the reactions on pyridinium ions shown in Scheme 6, the parent heteroarenes were employed in combination with formic acid to generate the protonated species *in situ* [18].

**Scheme 11:**
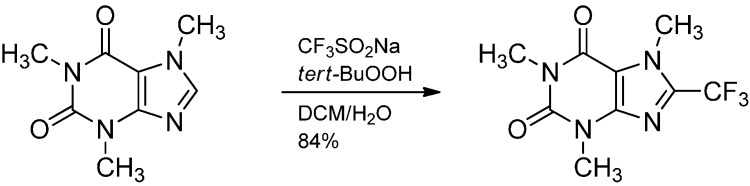
Trifluoromethylation of caffeine with the Langlois reagent.

The trifluoromethylation of nitrogen heterocycles by trifluoromethyl radicals generated *in situ* from sodium trifluoromethanesulfinate (CF_3_SO_2_Na, Langlois reagent) and *tert*-butylhydroperoxide has been reported by Baran in 2011 [32]. Here, a *tert*-butoxyl radical generates a CF_3_SO_2_ radical, which fragments into SO_2_ and a CF_3_ radical. Various medicinally relevant heterocycles have been trifluoromethylated under these conditions, for example caffeine produced the 8-trifluoromethyl derivative in 84% (Scheme 11). Whether a hydrogen-bonded species acts as the radical acceptor in this case remains unclear, an effect of Lewis-acidic metal additives has been observed in the same reaction on pyridines. The corresponding zinc trifluoromethanesulfinate gives superior results compared to the sodium salt [33].

**Scheme 12:**
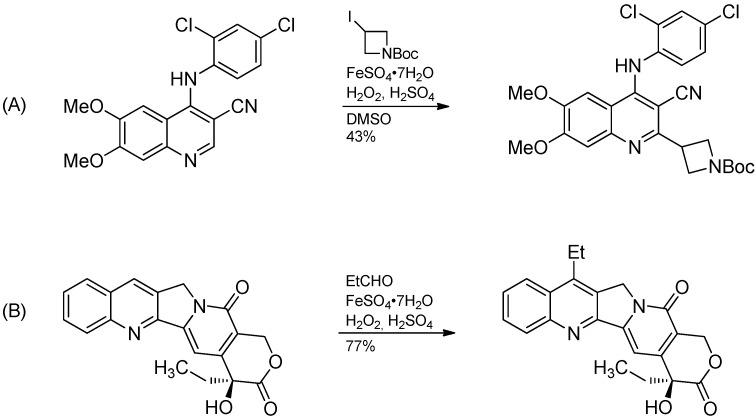
(**A**) Introduction of an azetidin-3-yl group. (**B**) Ethylation of camptothecin.

In summary, the Minisci reaction is a versatile and useful transformation for the late-stage functionalization of nitrogen heterocycles due to the functional group tolerance and the observed selectivity. It can be used for the structural diversification of medically relevant molecules without the need for protection/deprotection sequences as demonstrated by an impressive example, the regioselective direct ethylation of the chemotherapeutic agent camptothecin in 77% yield (Scheme 12B) [9]. The intermediate propionyl radicals generated by H-abstraction from propionic aldehyde decarbonylate prior the attack of the substrate to produce ethyl radicals.

**Figure 5:**
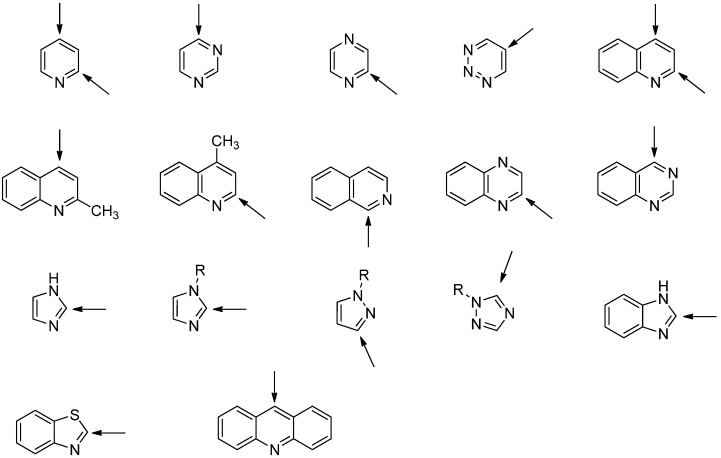
Overview for preferred positions of radical attack to the protonated forms of various heterocyclic bases.

The innate regioselectivity for the attack of C-centered radicals to the protonated forms of different heterocyclic bases is compiled in Figure 5.

### 2.3. Addition to Quaternized Pyridines and Heterocycles

**Scheme 13:**
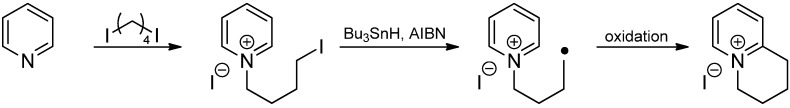
Synthesis of a bicylic ring system by a radical cyclization of an *N*-alkylpyridinium salt.

The construction of polynuclear heterocyclic systems from precursors carrying both a halogen atom and a pyridinium moiety has been demonstrated in several instances. The synthesis of 1,2,3,4-tetrahydroquinolizinium iodide by a radical addition to a quaternized pyridinium salt using tributyltin hydride for the dehalogenation was first reported by Murphy [34] in 1990 and has been expanded to [5,6] and [6,7]-membered ring systems as well (Scheme 13) [35].

**Scheme 14:**
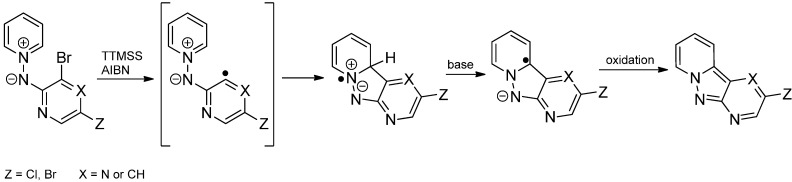
Aryl radical addition to an electron-deficient pyridinium fragment.

An intramolecular radical addition of an aryl radical to an electron-deficient pyridinium fragment has been described in 2002 by Alvarez-Builla within the formation of pyridopyrazole derivatives (Scheme 14) [36]. A 5-exo/endo-trig cyclisation is proposed to afford a tricyclic zwitterionic radical. Deprotonation and oxidation furnishes the tricyclic compounds in 20%–56% yield.

**Scheme 15:**
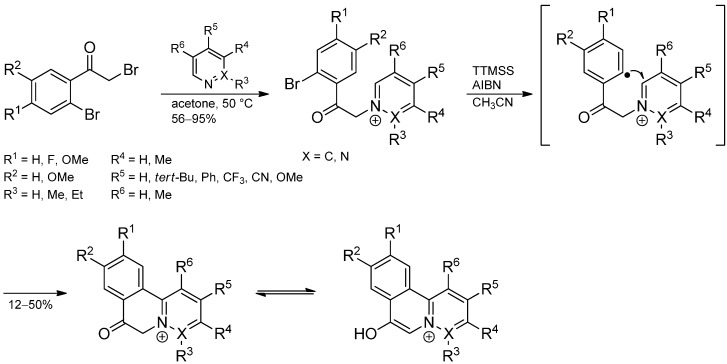
Intramolecular aryl radical addition to an electron-deficient pyridinium fragment.

The reaction of substituted α,2-dibromoacetophenones with pyridines affords the corresponding pyridinium salts which undergo a similar radical cyclization to furnish tricyclic pyridinium salts. The method was extended to a broad range of azinium salts (e.g., isoquinolinium, quinolinium, phenantridinium, phthalazin-2-ium) which afford the related polycyclic heteroaromatic compounds in moderate yields (Scheme 15) [37]. The presence of electron-withdrawing substituents in the azinium moiety results in a decreased yield whereas electron-releasing groups on both aromatic rings facilitate the arylation process.

## 3. Addition to Aliphatic Iminium Ions

### 3.1. The Knabe Rearrangement

**Scheme 16:**
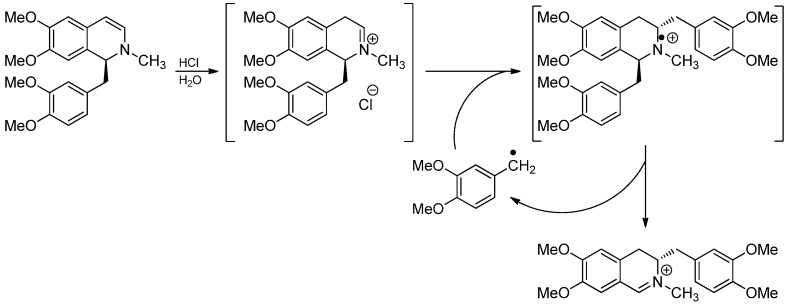
Radical mechanism of the Knabe rearrangement postulated by Langhals and Rüchardt.

In 1963, Knabe and coworkers reported the stereospecific rearrangement of 1,2-dihydroisoquinolines under acidic conditions (Scheme 16) [38,39]. Initially, an anionic mechanism had been proposed [40,41,42] until Langhals and Rüchardt [43] reported evidence for a free radical chain reaction involving the addition of *in situ* generated benzylic radicals to iminium ions in 1984. The negative results of EPR and CIDNP were explained by efficient chain reactions. Proof for a free radical chain reaction was the formation of corresponding bibenzyls [41] as side products and especially the inhibition by oxygen, tribromoacetic acid, 3-cyanopyridine or diethyl ether [43]. The initiation mechanism of the radical chain reaction is still ambiguous. However, autoxidation to form labile hydroperoxides may be responsible.

**Scheme 17:**
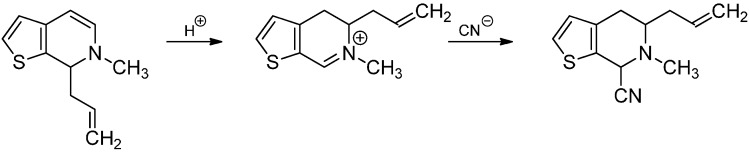
Knabe rearrangement with 7-allyl-6,7-dihydrothieno[2,3-c]pyridine.

The Knabe rearrangement is not limited to a benzyl substituent on C‑1 or on the isoquinoline moiety. It has also been observed with naphthalen-1-ylmethyl-, naphthalen-2-ylmethyl- or anthracen-9-ylmethyl substituents [44]. Furthermore, unsaturated aliphatic side chains like allyl [45], phenylpropargyl [46] or cinnamyl [47] were rearranged successfully as well. Instead of isoquinolines 7-allyl-6,7-dihydrothieno[2,3-c]pyridine could also be used as a substrate (Scheme 17) [48].

**Scheme 18:**
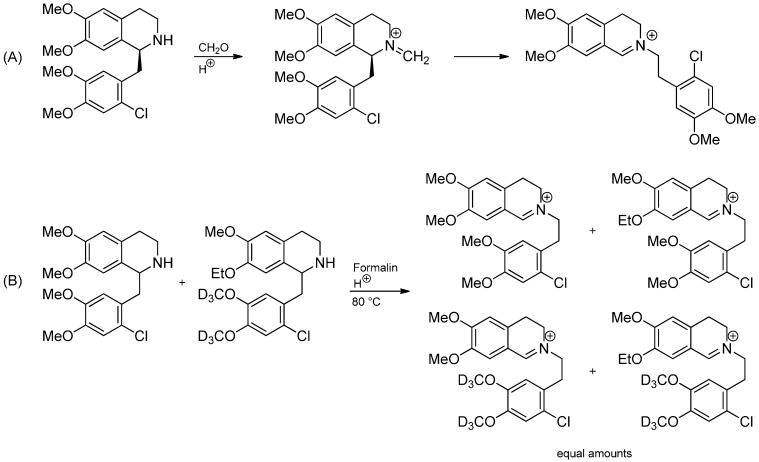
(**A**) 1,3-benzyl migration in 1-benzyl-2-methylene-1,2,3,4-tetrahydroisoquinolin-2-ium salts; (**B**) Cross-over experiment.

A related rearrangement has been observed in 1-benzyl-2-methylene-1,2,3,4-tetrahydroisoquinolin-2-ium salts by Blank *et al.* (Scheme 18) [49]. A free radical mechanism was supported by the observation of reaction inhibition by iodine, cuprous chloride or TEMPO and rate acceleration by radical initiators like dibenzoyl peroxide. Besides, the proposed mechanism was supported by a DFT calculation and cross-over experiments pointed out that the reaction proceeds intermolecularly. In contrast to the Knabe rearrangement however, no inhibition by oxygen and no interference by radical acceptors like ethyl acrylate could be observed, requiring very favorable kinetics of the addition of the benzylic radical to the intermediate methyleneiminium ion.

### 3.2. Radical Addition to “Cation Pool” Species

**Scheme 19:**
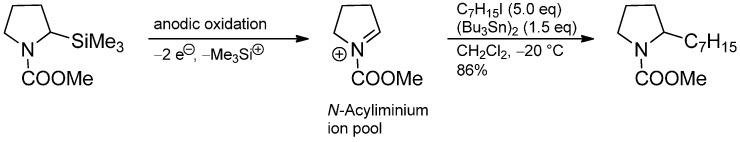
Distannane mediated alkylation of *N*-acyliminium ions.

Yoshida and coworkers reported the generation of an *N*-acyliminium “cation pool” by anodic oxidation of carbamates at low-temperatures in 1999 [50]. Besides the trapping of the cation with various nucleophiles [51], free radical additions to the iminium ion were investigated commencing in 2005 [52,53]. In this context, the distannane mediated alkylation with alkyl iodides has been reported (Scheme 19).

**Scheme 20:**
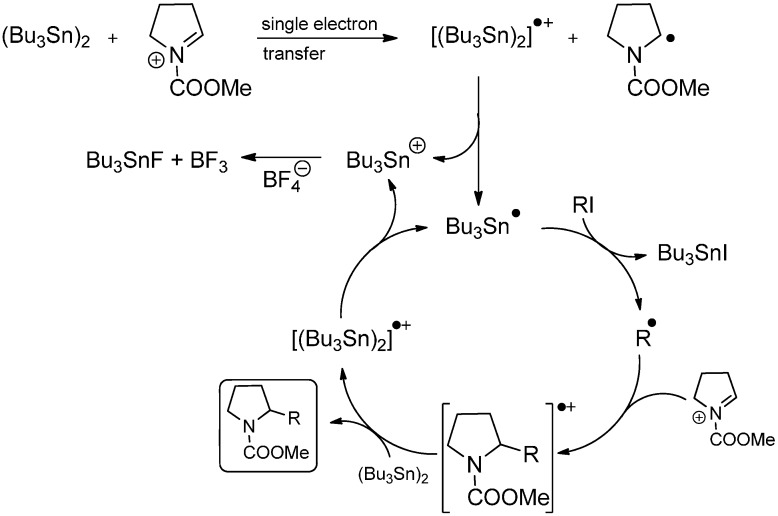
Proposed mechanism for the distannane mediated alkylation of *N*-acyliminium ions.

The proposed mechanism for this reaction is initiated by a single-electron transfer (SET) from hexabutyldistannane to the *N*-acyliminium ion. The generated radical cation induces the formation of a tributylstannyl radical and a tributylstannyl cation under Sn–Sn-bond cleavage, with the latter one being trapped by tetrafluoroborate, the anion of the electrolyte used for electrolysis. Iodine abstraction by the tributylstannyl radical from the alkyl iodide affords an alkyl radical which adds to the *N*-acyliminium ion to form another radical cation. A subsequent electron transfer reaction with hexabutyldistannane furnishes the final product and the radical cation of hexabutyldistannane which closes the catalytic cycle (Scheme 20).

Various organic iodides have been successfully employed, resulting in yields of 35%–86%. Remarkably, the reaction with cyclopropylmethyl iodide leads to the desired product in moderate yield along with the ring-opened product. As the cyclopropylmethyl radical is known for its very fast ring-opening rate constant of 1.3 × 10^8^ s^−1^ [54], the rate of the radical addition to the very electron deficient *N*-acyliminium ion has to be of similar velocity. When the reaction is performed in absence of an organic iodide, the dimer of *N*-methoxycarbonylpyrrolidine is formed. When alkyl bromides were used, the reaction was rather slow and with alkyl chlorides, no product formation could be observed. Hence, 1-chloro-6-iodoheptane affords the chloroheptyl-substituted product in high selectivity.

**Scheme 21:**
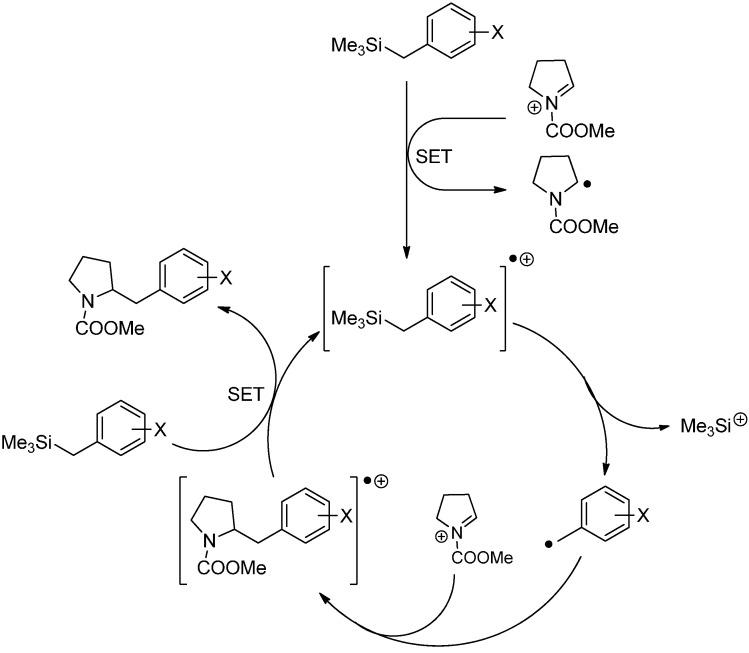
Proposed mechanism for the reaction of benzylsilanes with *N*-acyliminium ions.

A similar reaction has been reported for benzyl silanes [55]. In this case, a benzyl silane radical cation is formed first by a single electron transfer to the *N*-acyliminium ion. The radical cation decomposes to a silyl cation and a benzyl radical which attacks the *N*-acyliminium ion. The generated carbamate radical cation is reduced by benzyl silane and furnishes the desired product and a benzyl silane radical cation (Scheme 21). When benzyl silanes with a high oxidation potential are used, catalytic amounts of benzyl stannane can propagate an effective chain reaction [55]. Furthermore, arylthiomethylsilanes and aryloxymethylsilanes can be used as the precursor for the radical addition to *N*-acyliminium ions [56].

### 3.3. Addition to Activated Imines and Imine Derivatives

Nucleophilic addition of organometallic reagents to imines or derivatives bearing an acidic α-hydrogen fails and proton abstraction occurs instead. This can be avoided by addition of free radicals to the C=N bond. These reactions have been dormant for a long time because of the poor electrophilicity of the imino group, but in the last decades, radical additions to imines have been investigated with increasing attention. The radical addition to imines and derivatives has been summarized in excellent articles by Friestad [57], Naito [58], Punta [59], and Miyabe [60]. Mechanistically, iminium species can be proposed to be the actual radical acceptors since Lewis acids are usually added to improve the results. Intramolecular attack from radicals to imines is a synthetically valuable reaction [57,61,62,63], although the intermolecular reaction is less known [64].

#### 3.3.1. Porta Reaction

**Scheme 22:**
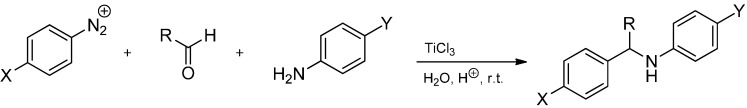
Arylative amination of aldehydes promoted by aqueous titanium trichloride.

The intermolecular radical addition to activated imines using a TiCl_3_/ArN_2_^+^ system was first reported by Clerici and Porta [65] in 1990 and has been reviewed by Pastori *et al.* [59] in 2009 and by Punta and co-workers in 2012 [66]. Here, a primary aromatic amine, an aldehyde and an arenediazonium salt were converted to a secondary amine in a three-component reaction (Scheme 22).

**Scheme 23:**
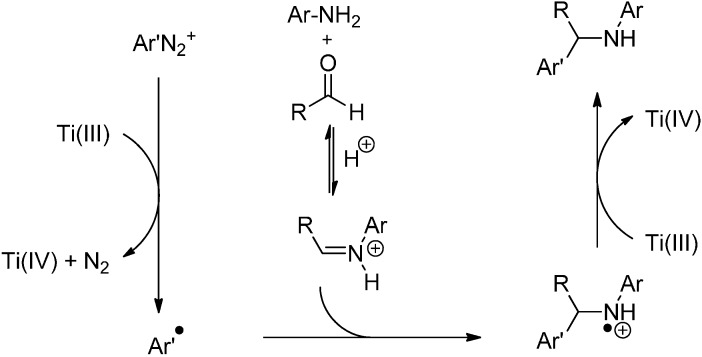
Proposed mechanism for the arylative amination promoted by TiCl_3_/PhN_2_^+^.

First, decomposition of the arenediazonium salt induced by SET from Ti^3+^ affords the aryl radical which presumably adds to the protonated aldimine formed *in situ* from the aniline and the aldehyde under the strongly acidic (AcOH/aq. HCl) reaction conditions. The resulting amine radical cation is further reduced to the amine by another equivalent of Ti^3+^ (Scheme 23) [66]. Hence, Ti^3+^ acts as a radical initiator as well as a radical terminator in the final reduction [67].

**Scheme 24:**
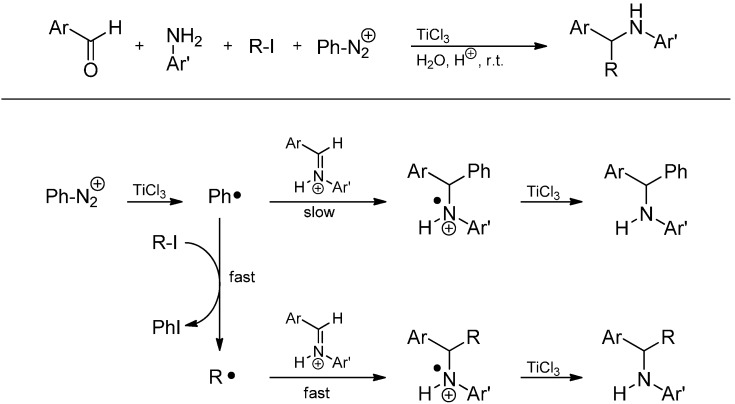
Alkylative amination of aldehydes in a four-component reaction.

In 2005, the reaction has been expanded by adding an alkyl iodide as a fourth reaction component [67]. Because of a fast iodine-atom abstraction from the alkyl iodide by the phenyl radical and the higher nucleophilicity of alkyl radicals compared to the phenyl radical, alkylation of the intermediate iminium ion occurs smoothly (Scheme 24). Various alkyl iodides (methyl, heptyl, isopropyl, cyclohexyl and benzyl) afford the secondary amine in fair to good isolated yields (50%–82%). However, the addition of the *tert*-butyl radical failed because of steric hindrance.

The same TiCl_3_/PhN_2_^+^-system has been used for radical addition of ethers to aldimines [68]. The produced phenyl radical generates an ether radical which, due to its high-lying SOMO, smoothly adds to the protonated C=N bond. The reaction was applicable for formaldimine, imines of enolizable aliphatic aldehydes, as well as aromatic aldehydes. THF, diethyl ether and 1,4-dioxane have been used as the ether component but in the case of THF, the competitive abstraction of the β-hydrogen has been observed as well (up to 10%).

**Scheme 25:**
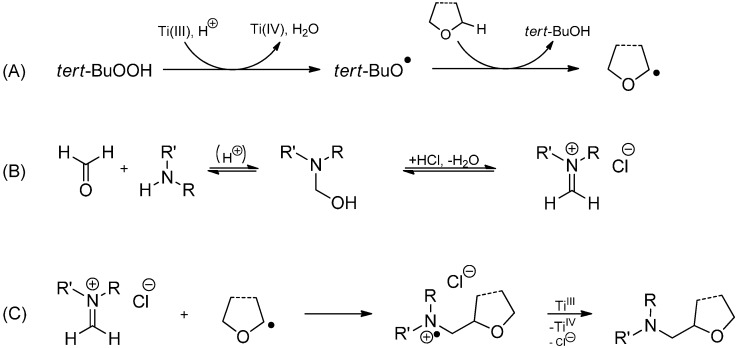
Aminomethylation of ethers. (**A**) Generation of the ether radical; (**B**) Formation of the iminium ion; (**C**) Radical addition and reduction to the 1,2-amino alcohol.

This radical aminomethylation has been optimized by switching to an acidic aqueous TiCl_3_/*tert*-BuOOH-system. Therein, a *tert*-butoxyl radical is produced instead of the phenyl radical which selectively abstracts an α-hydrogen from the ether (Scheme 25). As an alternative procedure, the Ti(III) species can be regenerated by reduction with zinc [69].

**Scheme 26:**
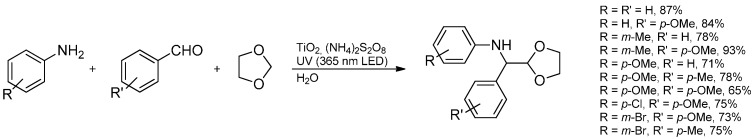
Aminomethylation of ethers using TiO_2_/(NH_4_)_2_S_2_O_8_ and UV light.

A variety of amines and THF, 1,4-dioxane or diethyl ether yielded the 1,2-aminoalcohols in 40%–88% isolated yield when formaldehyde was used. Changing to aliphatic or aromatic aldehydes resulted in 63%–80% yields of the desired products, presumed that a primary amine is used. Other amines failed to give the reaction, presumably due to steric factors [70]. Radical addition of cyclic ethers to the C=N bond can also be initiated with the TiO_2_/(NH_4_)_2_S_2_O_8_ system under UV-irradiation as reported by Shi and coworkers in 2013 (Scheme 26) [71]. Here, the acidic ammonium peroxydisulfate acts as a proton source to activate the basic imine. Earlier reports of TiO_2_/sunlight induced coupling reactions to heterocyclic bases date back to 2003 [72,73].

**Scheme 27:**
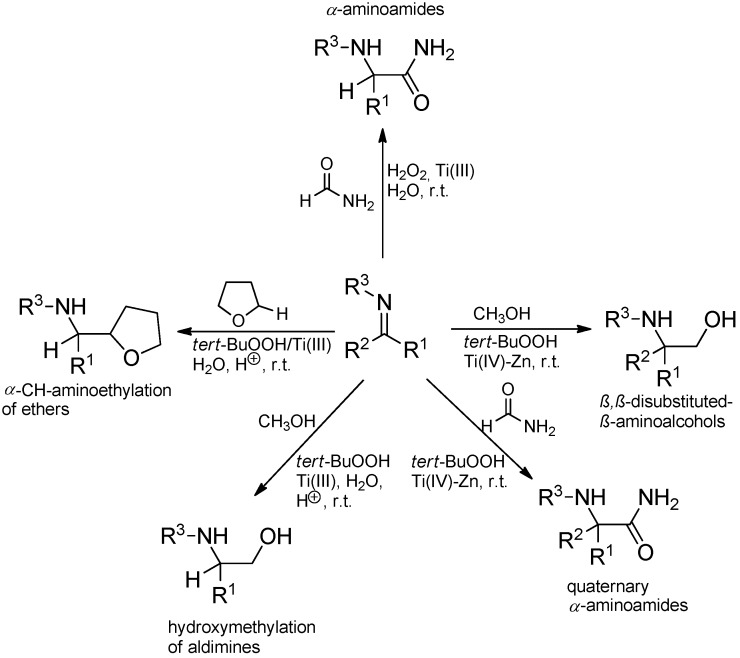
Radical additions to imines triggered by the Ti(III)/ROOH system (Porta reaction).

Further investigations allowed to extend the scope of the reaction to the addition of different nucleophilic radicals to the C=N bond so that e.g. hydroxymethylation or carbamoylation of aldimines or ketimines became feasible (Scheme 27) [66,74,75,76,77,78].

#### 3.3.2. Intermolecular Radical Addition to Imines Initiated by Dimethylzinc-Air

**Scheme 28:**
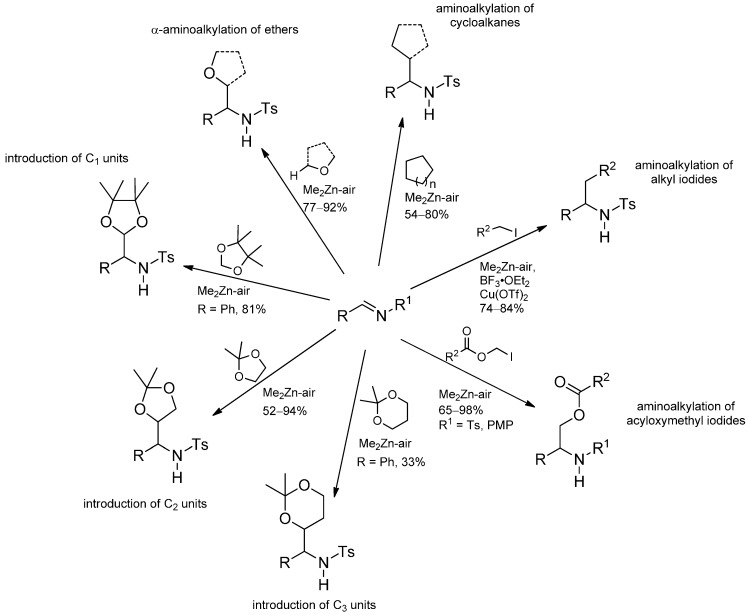
Radical addition to imines initiated by dimethylzinc-air.

Radical additions to glyoxylate imines producing α-amino acids using the Et_2_Zn/O_2_ system in combination with iodoalkanes had been reported by the Bertrand group in the late 1990’s [79,80]. The radical addition to imines has also been reported by Tomioka and coworkers. Methyl radicals generated from Me_2_Zn/O_2_ attack the C=N bond which is exclusively (as in the case of N-aryl substituents) or additionally (as in the case of N-tosyl groups) activated by coordination of the Lewis-acidic zinc species. Ethers [64,81,82,83], cycloalkanes [84], primary alkyl iodides [85] and acyloxymethyl iodides [86] can act as radical precursors [59]. Furthermore, oxygen functionalized C_1_, C_2_ and C_3_ units [87] were introduced to the imines. The versatility of the Me_2_Zn/O_2_-initiated radical addition is summarized in Scheme 28.

#### 3.3.3. Intermolecular Radical Addition to Imines Initiated by Trialkylboranes

**Scheme 29:**
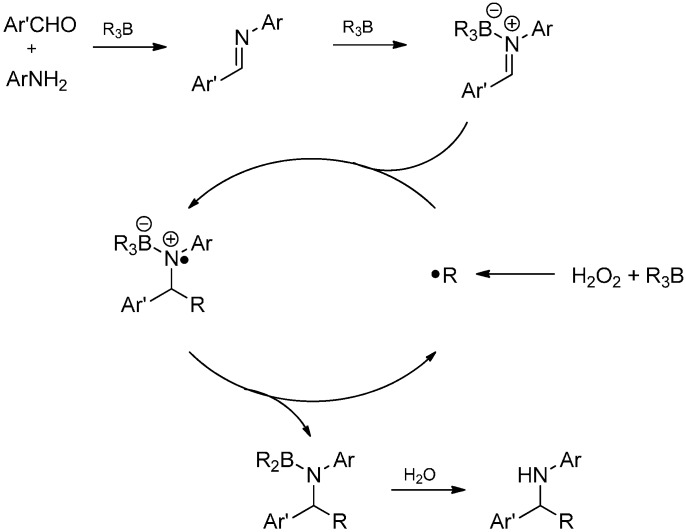
Proposed mechanism for the alkylative amination reaction with trialkylboranes.

A three component reaction with an aldehyde, an amine and a trialkylborane as a multifunctional reagent has also been reported [88]. The borane exhibits a triple role in this reaction: it serves as a Lewis acid catalyst and facilitates imine formation and further activates the imine towards radical addition by coordination. Moreover, it delivers the alkyl radical, in fact the nucleophilic species, which attacks the imine to afford the alkylated amines (Scheme 29).

**Scheme 30:**
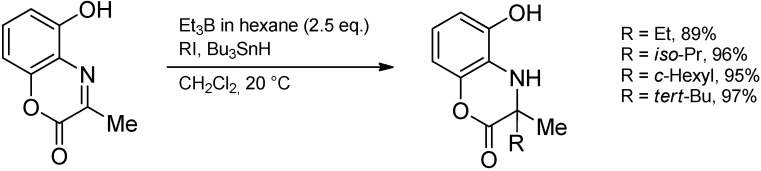
Alkyl radical addition to ketimines with a 2-phenolic hydroxyl group.

The reaction is applicable to a broad spectrum of starting materials. Aromatic, heteroaromatic, as well as enolizable aliphatic aldehydes can be combined with aromatic and aliphatic amines to furnish the desired products. The type of the alkyl side chain is determined by the trialkylborane used and includes primary as well as secondary alkyl or cycloalkyl. In the case of aliphatic amines, the imine activation by the trialkylborane is too feeble and addition of BF_3_·OEt_2_ is required. Under these reaction conditions, endocyclic imines like 3,4-dihydroisoquinolines are also converted. The alkylation of aldimines and ketimines having an *ortho*-phenolic hydroxyl group proceeds smoothly due to the effective activation of the C=N bond and additional stabilization of the intermediate aminyl radical by an intramolecular hydrogen bond (Scheme 30) [89].

#### 3.3.4. Intermolecular Radical Addition to Imine Derivatives

**Scheme 31:**
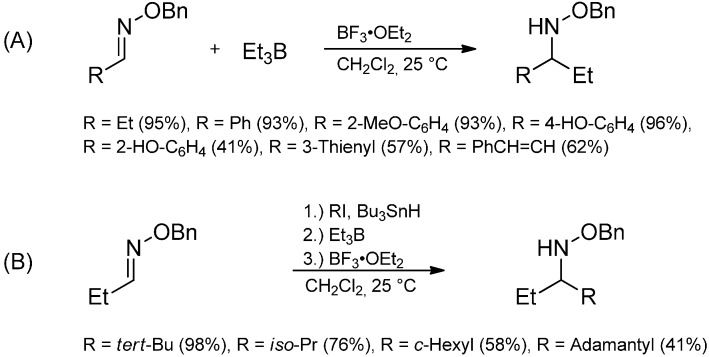
Radical attack to activated aldoxime ethers.

The radical additions described here can also be applied to imine derivatives like oxime ethers or hydrazones. Oxime ethers are excellent radical acceptors because their lone pair on the adjacent oxygen atom stabilizes the intermediate aminyl radical. Various *O*-benzyl aldoximes are ethylated by triethylborane under BF_3_·OEt_2_ catalysis and diverse types of radicals have been successfully applied (Scheme 31) [60,90,91,92].

**Scheme 32:**
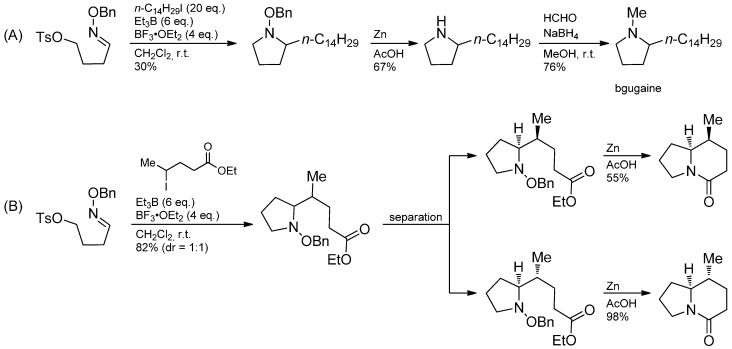
(**A**) Synthesis of bgugaine (**B**) Synthesis of a synthetic key intermediate of poison frog alkaloids of the indolizidine type.

A radical addition-nucleophilic ionic cyclization sequence of oxime ethers bearing a leaving group in the ω-position leads to pyrrolidines and piperidines. In this sequence, a carbon-centered radical attacks the oxime ether in the known fashion and the obtained *O*-benzylhydroxylamine cyclizes in an intramolecular alkylation. This methodology has been used in the total syntheses of bgugaine including subsequent cleavage of the oxime ether and reductive N-methylation (Scheme 32). Furthermore, a lactam moiety has been prepared, which is considered as a synthetic key intermediate of poison dart frog alkaloids [93,94,95] of the indolizidine type [96].

**Scheme 33:**
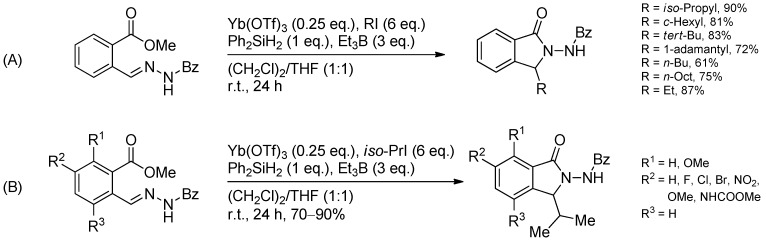
Radical addition-cyclization cascade with different alkyl iodides (**A**) and with various substituted hydrazones (**B**).

Another Lewis acid-catalyzed radical addition-cyclization cascade leads to 3-substituted isoindolin-1-one derivatives (Scheme 33). Here, different alkyl iodides have been used for the addition to the activated *N*-benzoyl hydrazone. Besides, various substituted hydrazones have been converted successfully to the respective imines [97]. However, low yields were observed in the presence of *ortho*-substituents (R^1^ = H, R^2^ = R^3^ = OMe).

**Scheme 34:**
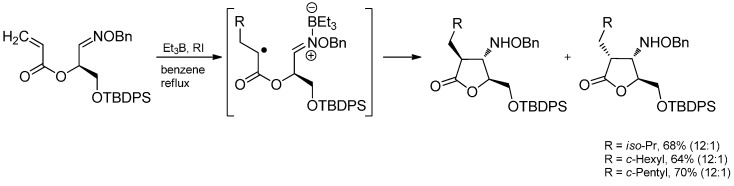
Radical addition-cyclization of oxime ethers.

The radical addition to oxime ethers can also occur intramolecularly after a radical addition to a Michael system, furnishing a carbon-centered radical which then readily attacks the activated oxime ether (Scheme 34). A high 1,2-stereoinduction was observed which can be ascribed to the A^1,3^-strain effect [98].

**Scheme 35:**
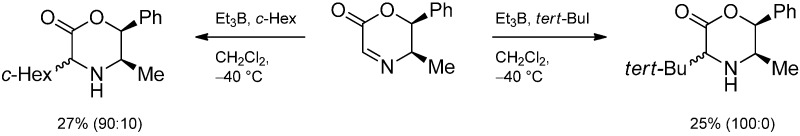
Diastereoselective radical addition to oxazinones.

**Scheme 36:**
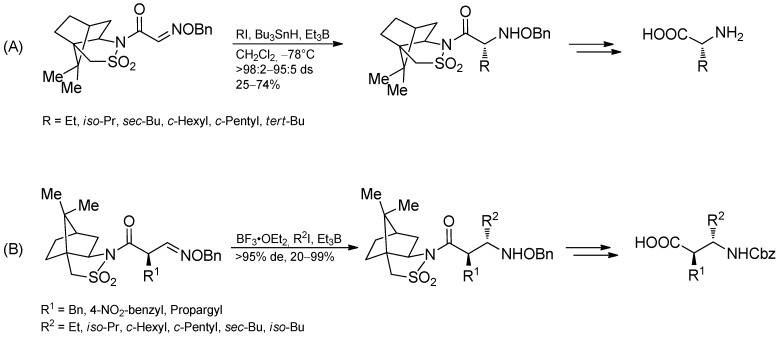
Diastereoselective radical addition-cyclization of oxime ethers to yield (**A**) α-amino acids or (**B**) β-amino acids.

The addition of alkyl radical to oxazinones gave moderate yields but again, high diastereoselectivities could be observed (Scheme 35) [99]. Oppolzer’s camphorsultam has been used as a chiral auxiliary in highly diastereoselective radical addition to glyoxylic oxime ethers and oxime ethers to yield α- or β-amino acids after further transformations (Scheme 36) [60,100,101,102]. Due to the lower reactivity of oxime ethers compared to the glyoxylic oxime ethers, an additional activation of the C=N-bond with BF_3_·OEt_2_ was required.

**Scheme 37:**
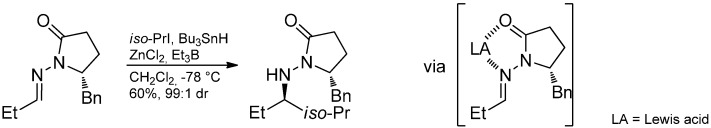
Radical addition-cyclization of oxime ethers.

Alternatively, Lewis acid-activated chiral *N*-acylhydrazones were used by the Friestad group and the radical addition proceeded smoothly and with excellent selectivity (Scheme 37) [60,103,104,105,106,107,108,109,110].

**Scheme 38:**
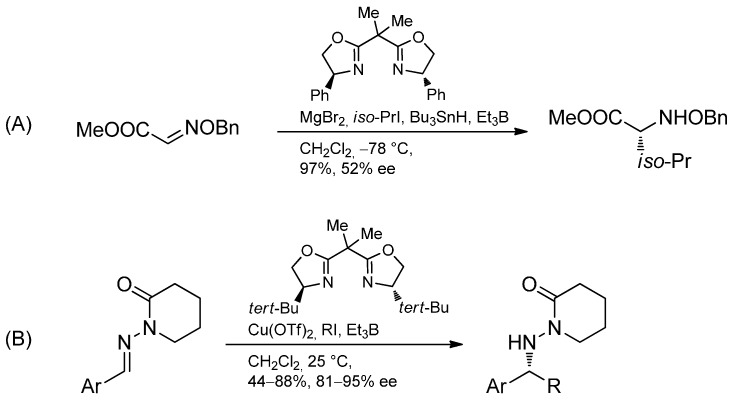
(**A**) First enantioselective radical addition to form a valine derivative. (**B**) Enantioselective addition to *N*-acyl hydrazones.

The first enantioselective free radical addition to activated C=N bonds was reported by Naito and Miyabe [60,111]. A chiral Lewis acid formed from the BOX-ligand (R)-(+)-2,2'-isopropylidenebis(4-phenyl-2-oxazoline) and MgBr_2_ allowed the preparation of a valine derivative with 52% ee. A further development by the Friestad group involving *N*-acylhydrazones in combination with a copper-based chiral Lewis acid led to the addition of various alkyl radicals to aromatic hydrazones with high levels of enantioselectivity (Scheme 38) [112].

### 3.4. Iminium Ions in Photoredox Chemistry

**Scheme 39:**
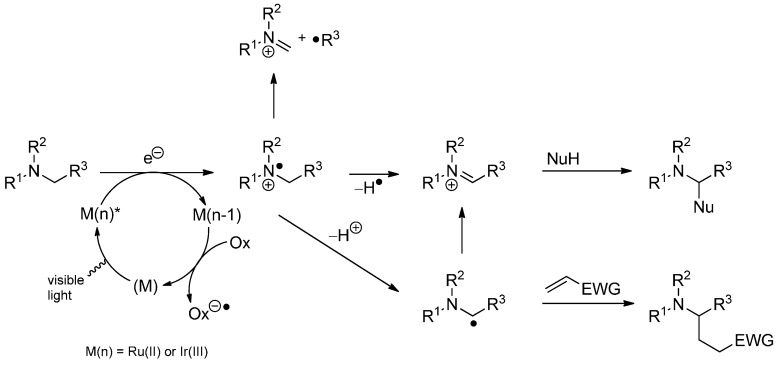
Generation of amine radical cations and their mode of reactivity.

Tertiary amines can be oxidized to radical cations by the action of photocatalysts including ruthenium and iridium polypyridyl complexes in a single electron transfer (SET) process. The generated odd electron species can react in different ways. Besides the unproductive backward reaction, a C–C bond fragmentation can occur to furnish an iminium ion and a neutral radical. Alternatively, deprotonation affords an α-amino radical, which can react with an olefin or can be converted to an iminium ion by another single electron oxidation. The same iminium ion can also be obtained by homolytic hydrogen abstraction from the initially generated amine radical cation. The resulting iminium ion reacts with nucleophiles to α-substituted amines and a radical addition is also possible (Scheme 39) [113].

**Scheme 40:**
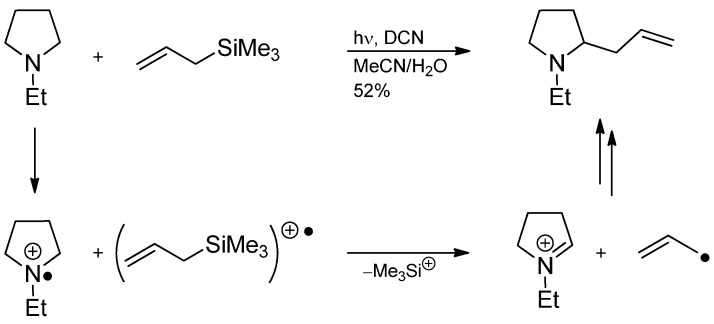
Photoredox allylation of 1-ethylpyrrolidine with allyltrimethylsilane.

The radical addition to an iminium ion in a photoredox reaction had been discussed by Pandey *et al.* in 1992. 1-Ethylpyrrolidine and allyltrimethylsilane react with dicyanonaphthalene (DCN) and light irradiation to 2-allyl-1-ethylpyrrolidine (Scheme 40). Therein, the amine is oxidized to the iminium ion and is attacked by an allyl radical generated from the silane in a separate SET/fragmentation process [114,115].

**Scheme 41:**
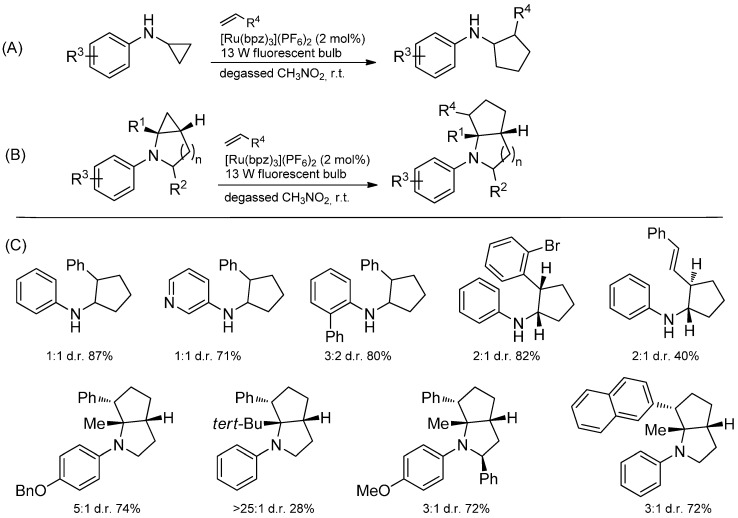
(**A**) Photooxidation of monocyclic *N*-cyclopropylanilines; (**B**) Photooxidation of bicyclic *N*-cyclopropylanilines; (**C**) Examples of [3+2]-annulated products.

**Scheme 42:**
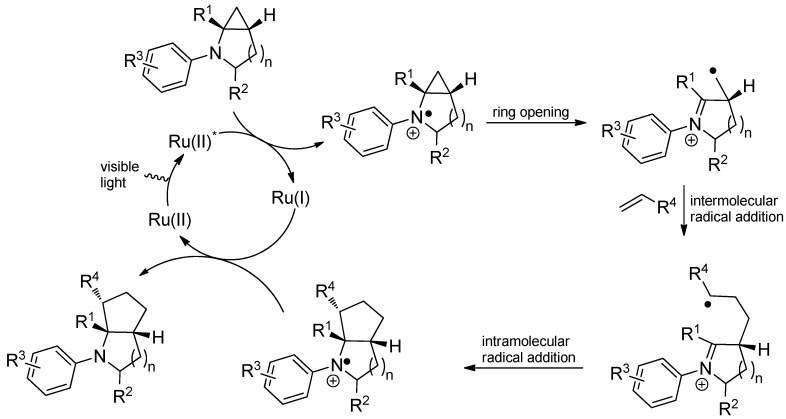
Proposed mechanism for intermolecular [3+2] annulation of *N*-cyclopropylamines with olefins.

The ruthenium-catalyzed photooxidation of mono- and bicyclic *N*-cyclopropylanilines with alkenes demonstrates the modes of reactivity outlined in Scheme 41 and yields [3+2]-annulated products. First, the amine is oxidized by SET to the radical cation which undergoes a rapid opening assisted by the ring strain. The obtained neutral radical reacts with an added olefin and the newly generated radical attacks the iminium ion to furnish a less strained amine radical cation which in turn is reduced by the photocatalyst to the [3+2]-annulated product (Scheme 42) [113].

## 4. Conclusions

In summary, the strong interaction of the SOMO of carbon-centered radicals with the low-lying LUMO of a positively charged C=N double bond, as present in iminium ions as well as protonated or N-alkylated nitrogen heterocycles, results in fast and often highly selective addition reactions. This elementary process is the basis of a variety of several preparatively useful C–C bond forming reactions that generally do not require the use of protecting groups and can, for example, be used for the post-functionalization of complex substrates. Due to its efficiency, the radical addition to organic cations is likely to be part of further interesting developments in preparative free radical chemistry.

## References

[1] Fleming I. (2010). Molecular Orbitals and Organic Chemical Reactions, Reference Edition.

[2] Minisci F., Bernardi R., Bertini F., Galli R., Perchinummo M. (1971). Nucleophilic character of alkyl radicals—VI: A new convenient selective alkylation of heteroaromatic bases. Tetrahedron.

[3] Halland N., Jorgensen K.A. (2001). Intermolecular addition of alkyl radicals to imines in the absence and in the presence of a Lewis acid. J. Chem. Soc..

[4] Frisch M.J., Trucks G.W., Schlegel H.B., Scuseria G.E., Robb M.A., Cheeseman J.R., Scalmani G., Barone V., Mennucci B., Petersson G.A. (2009). Gaussian 09.

[5] Wu Y.D., Lai D.K.W., Houk K.N. (1995). Transition structures of hydride transfer-reactions of protonated pyridinium Ion with 1,4-dihydropyridine and protonated nicotinamide with 1,4-dihydronicotinamide. J. Am. Chem. Soc..

[6] Foresman J.B., Frisch A. (1996). Exploring Chemistry with Electronic Structure Methods.

[7] Augood D.R., Williams G.H. (1957). Homolytic aromatic arylation. Chem. Rev..

[8] Gomberg M., Bachmann W.E. (1924). The synthesis of biaryl compounds by means of the diazo reaction. J. Am. Chem. Soc..

[9] Duncton M.A.J. (2011). Minisci reactions: Versatile CH-functionalizations for medicinal chemists. Med. Chem. Comm..

[10] Lynch B.M., Chang H.S. (1964). Free-radical phenylations of 1-methyldiazoles. Tetrahedron Lett..

[11] Lynch B.M., Chang H.S. (1964). Concentration-dependent orientations in free-radical phenylations of heteroaromatic compounds. Tetrahedron Lett..

[12] Dou H.J.M., Lynch B.M. (1965). Selective free-radical phenylations: Nitrogen-heteroaromatic compounds in acidic media. Tetrahedron Lett..

[13] Dou H.J.M. (1966). Méthylation radicalaire du thiazole et de ses derivés méthyles en milieu acétique. Bull. Soc. Chim. Fr..

[14] Dou H.J.M., Lynch B.M. (1966). Phénylation radicalaire sélective en milieu acide-composés hétéroaromatiques azotes I. Methode et résultats expérimentaux. Bull. Soc. Chim. Fr..

[15] Dou H.J.M., Lynch B.M. (1966). Phénylation radicalaire sélective en milieu acide-composés hétéroaromatiques azotes 2. Traitement théorique des résultats expérimentaux et discussion. Bull. Soc. Chim. Fr..

[16] Punta C., Minisci F. (2008). Minisci reaction: A Friedel-Crafts type process with opposite reactivity and selectivity. Selective homolytic alkylation, acylation, carboxylation and carbamoylation of heterocyclic aromatic bases. Trends Heterocycl. Chem..

[17] Seiple I.B., Su S., Rodriguez R.A., Gianatassio R., Fujiwara Y., Sobel A.L., Baran P.S. (2010). Direct C-H arylation of electron-deficient heterocycles with arylboronic acids. J. Am. Chem. Soc..

[18] Xue D., Jia Z.-H., Zhao C.-J., Zhang Y.-Y., Wang C., Xiao J. (2014). Direct arylation of *N*-heteroarenes with aryldiazonium salts by photoredox catalysis in water. Chem. Eur. J..

[19] Minisci F., Vismara E., Fontana F. (1989). Recent developments of free-radical substitutions of heteroaromatic bases. Heterocycles.

[20] Minisci F., Fontana F., Vismara E. (1990). Substitutions by nucleophilic free-radicals—A new general reaction of heteroaromatic bases. J. Heterocycl. Chem..

[21] Harrowven D.C., Sutton B.J., Coulton S. (2002). Intramolecular radical additions to quinolines. Tetrahedron.

[22] Harrowven D.C., Sutton B.J., Coulton S. (2003). Intramolecular radical additions to pyridines. Org. Biomol. Chem..

[23] Harrowven D.C., Sutton B.J. (2004). Radical additions to pyridines, quinolines and isoquinolines. Prog. Heterocycl. Chem..

[24] Bowman W.R., Storey J.M.D. (2007). Synthesis using aromatic homolytic substitution-recent advances. Chem. Soc. Rev..

[25] O’Hara F., Blackmond D.G., Baran P.S. (2013). Radical-based regioselective C-H functionalization of electron-deficient heteroarenes: Scope, tunability, and predictability. J. Am. Chem. Soc..

[26] Begg C., Grimmett M., Yu-Man L. (1973). The synthesis of 2-alkyl- and 2-acyl-imidazoles by substitution methods. Aust. J. Chem..

[27] Jain R., Cohen L.A., El-Kadi N.A., King M.M. (1997). Regiospecific alkylation of histidine and histamine at C-2. Tetrahedron.

[28] Mahindra A., Jain R. (2012). Regiospecific direct C-H arylation at the 2-position of L-histidine using arylboronic acids. Synlett.

[29] Hansen K.B., Springfield S.A., Desmond R., Devine P.N., Grabowski E.J.J., Reider P.J. (2001). Radical alkylation of N-alkyl 1,2,4-triazoles. Tetrahedron Lett..

[30] Joule J.A., Mills K. (2013). Heterocyclic Chemistry.

[31] Nagata K., Itoh T., Okada M., Takahashi H., Ohsawa A. (1991). Radical carbamoylation of 1,2,3-triazinium 2-dicyanomethylides. Heterocycles.

[32] Ji Y., Brueckl T., Baxter R.D., Fujiwara Y., Seiple I.B., Su S., Blackmond D.G., Baran P.S. (2011). Innate C-H trifluoromethylation of heterocycles. Proc. Natl. Acad. Sci. USA.

[33] Fujiwara Y., Dixon J.A., O’Hara F., Funder E.D., Dixon D.D., Rodriguez R.A., Baxter R.D., Herle B., Sach N., Collins M.R. (2012). Practical and innate carbon-hydrogen functionalization of heterocycles. Nature.

[34] Murphy J.A., Sherburn M.S. (1990). Intramolecular addition of Free radicals to quaternised heterocyclic rings. Tetrahedron Lett..

[35] Murphy J.A., Sherburn M.S. (1990). Intramolecular free-radical substitution reactions of pyridinium rings: Efficient formation of [5,6]- and [6,7]-fused ring systems. Tetrahedron Lett..

[36] Nunez A., de Viedma A.G., Martinez-Barrasa V., Burgos C., Alvarez-Builla J. (2002). *N*-Azinylpyridinium *N*-aminides: An approach to pyrazolopyridines via an intramolecular radical pathway. Synlett.

[37] Castillo R.R., Burgos C., Vaquero J.J., Alvarez-Builla J. (2011). Radical intramolecular arylation of pyridinium salts: A straightforward entry to 7-hydroxypyrido[2,1-a]isoquinolinium salts. Eur. J. Org. Chem..

[38] Knabe J., Kubitz J., Ruppenthal A.N. (1963). Umlagerung von N-Methyl-1.2-dihydropapaverin mit verdünnten Säuren. Angew. Chem..

[39] Knabe J., Kubitz J. (1964). Über eine Umlagerung von N-Methyl-1,2-dihydropapaverin mit verdünnten Säuren. Arch. Pharm. Ber..

[40] Knabe J., Heckmann R. (1980). Dihydroisoquinoline rearrangement 27. Proposal of a new mechanism. Arch. Pharm..

[41] Kinsman R.G., Dyke S.F. (1979). Mechanism of the rearrangement of 1-benzyl-1,2-dihydroisoquinolines—Some criticisms answered. Tetrahedron.

[42] Powilleit H. (1969). Untersuchungen zum Mechanismus der 1,2-Dihydroisochinolin-Umlagerung. Ph.D. Dissertation.

[43] Langhals E., Langhals H., Ruchardt C. (1984). Evidence for a radical chain mechanism for the Knabe reaction of 1,2-dihydro-2-methylpapaverine. Chem. Ber..

[44] Knabe J., Grunewald F.J. (1987). Rearrangement of dihydroisoquinolines 38. Behavior of 1,2-dihydroisoquinolines with bulky C-1-substituents towards acids. Arch. Pharm..

[45] Sainsbury M., Brown D.W., Dyke S.F., Kinsman R.G., Moon B.J. (1968). 1,2-Dihydroisoquinolines—VIII: Rearrangement—II. Tetrahedron.

[46] Knabe J., Hanke B. (1987). Dihydroisoquinoline Rearrangement 39. 6,7-Dimethoxy-2-methyl-1-(phenylpropargyl)-1,2-dihydroisoquinoline. Arch. Pharm..

[47] Knabe J., Holtje H.D. (1969). Dihydroisoquinoline rearrangement 9: Vinylog principle in rearrangement of tertiary 1,2-dihydroisoquinolines. Tetrahedron Lett..

[48] Knabe J., Lorenz J. (1983). Dihydroisoquinoline rearrangement 34. 7-Allyl-6-methyl-6,7-dihydrothieno[2,3-C]pyridine. Arch. Pharm..

[49] Blank N., Straub B.F., Opatz T. (2011). 1,3-Benzyl migration in iminium ions: Evidence for a fast free-radical chain reaction. Eur. J. Org. Chem..

[50] Yoshida J., Suga S., Suzuki S., Kinomura N., Yamamoto A., Fujiwara K. (1999). Direct oxidative carbon-carbon bond formation-using the “cation pool” method. 1. Generation of iminium cation pools and their reaction with carbon nucleophiles. J. Am. Chem. Soc..

[51] Yoshida J., Ashikari Y., Matsumoto K., Nokami T. (2013). Recent developments in the “cation pool” method. J. Synth. Org. Chem. Jpn..

[52] Maruyama T., Suga S., Yoshida J.-I. (2005). Radical addition to “Cation Pool”. Reverse process of radical cation fragmentation. J. Am. Chem. Soc..

[53] Maruyama T., Suga S., Yoshida J.I. (2006). Distannane mediated reaction of N-acyliminium ion pools with alkyl halides. A chain mechanism involving radical addition followed by electron transfer. Tetrahedron.

[54] Maillard B., Forrest D., Ingold K.U. (1976). Kinetic applications of electron-paramagnetic resonance spectroscopy 27. Isomerization of cyclopropylcarbinyl to allylcarbinyl. J. Am. Chem. Soc..

[55] Maruyama T., Mizuno Y., Shimizu I., Suga S., Yoshida J.-I. (2007). Reactions of a N-acyliminium ion pool with benzylsilanes. Implication of a radical/cation/radical cation chain mechanism involving oxidative C-Si Bond Cleavage. J. Am. Chem. Soc..

[56] Suga S., Shimizu I., Ashikari Y., Mizuno Y., Maruyama T., Yoshida J. (2008). Electro-initiated coupling reactions of N-acyliminium ion pools with arylthiomethylsilanes and aryloxymethylsilanes. Chem. Lett..

[57] Friestad G.K. (2001). Addition of carbon-centered radicals to imines and related compounds. Tetrahedron.

[58] Miyabe H., Ueda M., Naito T. (2004). Carbon-carbon bond construction based on radical addition to C=N bond. Synlett.

[59] Pastori N., Gambarotti C., Punta C. (2009). Recent developments in nucleophilic radical addition to imines: The key role of transition metals and the new Porta radical-type version of the Mannich and Strecker reactions. Mini-Rev. Org. Chem..

[60] Miyabe H., Yoshioka E., Kohtani S. (2010). Progress in intermolecular carbon radical addition to imine derivatives. Curr. Org. Chem..

[61] Naito T. (1999). Heteroatom radical addition-cyclization and its synthetic application. Heterocycles.

[62] Fallis A.G., Brinza I.M. (1997). Free radical cyclizations involving nitrogen. Tetrahedron.

[63] Miyabe H., Ueda M., Nishimura A., Naito T. (2002). Indium-mediated intermolecular alkyl radical addition to electron-deficient C=N bond and C=C bond in water. Org. Lett..

[64] Yamada K., Fujihara H., Yamamoto Y., Miwa Y., Taga T., Tomioka K. (2002). Radical addition of ethers to imines initiated by dimethylzinc. Org. Lett..

[65] Clerici A., Porta O. (1990). Arylative amination of aldehydes promoted by aqueous titanium trichloride. Tetrahedron Lett..

[66] Rossi B., Prosperini S., Pastori N., Clerici A., Punta C. (2012). New advances in titanium-mediated free radical reactions. Molecules.

[67] Cannella R., Clerici A., Pastori N., Regolini E., Porta O. (2005). One-pot four-component reaction: Aqueous TiCl_3_/PhN_2_^+^-mediated alkyl radical addition to imines generated *in situ*. Org. Lett..

[68] Clerici A., Cannella R., Panzeri W., Pastori N., Regolini E., Porta O. (2005). TiCl_3_/PhN_2_^+^-mediated radical addition of ethers to aldimines generated *in situ* under aqueous conditions. Tetrahedron Lett..

[69] Prosperini S., Pastori N., Ghilardi A., Clerici A., Punta C. (2011). New domino radical synthesis of aminoalcohols promoted by TiCl_4_-Zn/*t*-BuOOH system: Selective hydroxyalkylation of amines in alcohol or in cyclic ether cosolvents. Org. Biomol. Chem..

[70] Clerici A., Cannella R., Pastori N., Panzeri W., Porta O. (2006). A free radical Mannich type reaction: Selective α-CH aminomethylation of ethers by Ti(III)/*t*-BuOOH system under aqueous acidic conditions. Tetrahedron.

[71] Zhang L., Deng Y.Q., Shi F. (2013). Light promoted aqueous phase amine synthesis via three-component coupling reactions. Tetrahedron Lett..

[72] Caronna T., Gambarotti C., Palmisano L., Punta C., Recupero F. (2003). Sunlight induced functionalisation of some heterocyclic bases in the presence of polycrystalline TiO_2_. Chem. Commun..

[73] Gambarotti C., Punta C., Recupero F., Caronna T., Palmisano L. (2010). TiO_2_ in organic photosynthesis: Sunlight induced functionalization of heterocyclic bases. Curr. Org. Chem..

[74] Clerici A., Ghilardi A., Pastori N., Punta C., Porta O. (2008). A new one-pot, four-component synthesis of 1,2-amino alcohols: TiCl_3_/*t*-BuOOH-mediated radical hydroxymethylation of imines. Org. Lett..

[75] Spaccini R., Ghilardi A., Pastori N., Clerici A., Punta C., Porta O. (2010). Efficient radical domino approach to β-aminoalcohols from arylamines and alcohols triggered by Ti(III)/*t*-BuOOH. Tetrahedron.

[76] Pastori N., Greco C., Clerici A., Punta C., Porta O. (2010). Free-radical addition to ketimines generated *in situ*. New one-pot synthesis of quaternary α-aminoamides promoted by a H_2_O_2_/TiCl_4_-Zn/HCONH_2_ System. Org. Lett..

[77] Rossi B., Pastori N., Clerici A., Punta C. (2012). Free-radical hydroxymethylation of ketimines generated *in situ*: A one-pot multicomponent synthesis of β,β-disubstituted-β-aminoalcohols. Tetrahedron.

[78] Cannella R., Clerici A., Panzeri W., Pastori N., Punta C., Porta O. (2006). Free-radical version of the strecker synthesis of α-aminoamides promoted by aqueous H_2_O_2_/TiCl_3_/HCONH_2_ system. J. Am. Chem. Soc..

[79] Bertrand M.P., Feray L., Nouguier R., Perfetti P. (1999). Diethylzinc mediated radical additions to glyoxylate imines. Synlett.

[80] Bertrand M.P., Feray L., Nouguier R., Perfetti P. (1999). Diethylzinc: A chain-transfer agent in intermolecular radical additions. A parallel with triethylborane. J. Org. Chem..

[81] Yamada K., Yamamoto Y., Tomioka K. (2003). Initiator-dependent chemoselective addition of THF radical to aldehyde and aldimine and its application to a three-component reaction. Org. Lett..

[82] Yamamoto Y., Maekawa M., Akindele T., Yamada K., Tomioka K. (2005). Dimethylzinc-initiated radical reaction of cyclic ethers with arylamines, alkoxyamines, and dialkylhydrazines. Tetrahedron.

[83] Yamada K., Umeki H., Maekawa M., Yamamoto Y., Akindele T., Nakano M., Tomioka K. (2008). Conjugate addition reaction of THF-2-yl radical with α,β-unsaturated N-tosyl imines using a dimethylzinc-air initiator. Tetrahedron.

[84] Yamada K., Yamamoto Y., Maekawa M., Chen J.B., Tomioka K. (2004). Direct aminoalkylation of cycloalkanes through dimethylzinc-initiated radical process. Tetrahedron Lett..

[85] Yamada K., Yamamoto Y., Maekawa M., Akindele T., Umeki H., Tomioka K. (2006). Tin-free intermolecular addition of primary alkyls to imines via the dimethylzinc-air radical process. Org. Lett..

[86] Yamada K., Nakano M., Maekawa M., Akindele T., Tomioka K. (2008). Tin-free radical addition of acyloxymethyl to imines. Org. Lett..

[87] Yamada K.I., Yamamoto Y., Maekawa M., Tomioka K. (2004). Introduction of functionalized C1, C2, and C3 units to imines through the dimethylzinc—Air-initiated radical addition. J. Org. Chem..

[88] Valpuesta M., Munoz C., Diaz A., Torres G., Suau R. (2010). Multicomponent C-alkylation reactions of aromatic aldimines with trialkylboranes reagents. Eur. J. Org. Chem..

[89] Miyabe H., Yamaoka Y., Takemoto Y. (2006). Reactive ketimino radical acceptors: Intermolecular alkyl radical addition to imines with a phenolic hydroxyl group. J. Org. Chem..

[90] Miyabe H., Shibata R., Ushiro C., Naito T. (1998). Carbon-carbon bond formation via intermolecular carbon radical addition to aldoxime ethers. Tetrahedron Lett..

[91] Miyabe H., Shibata R., Sangawa M., Ushiro C., Naito T. (1998). Intermolecular alkyl radical addition to the carbon-nitrogen double bond of oxime ethers and hydrazones. Tetrahedron.

[92] Miyabe H., Yamakawa K., Yoshioka N., Naito T. (1999). Alkylative amination of aldehydes via carbon-carbon bond formation based on radical addition to carbon-nitrogen double bond. Tetrahedron.

[93] Song Y.C., Okamoto S., Sato F. (2002). A concise asymmetric synthesis of 5,8-disubstituted indolizidine alkaloids. Total synthesis of (−)-indolizidine 209B. Tetrahedron Lett..

[94] Aube J., Rafferty P.S., Milligan G.L. (1993). Application of the intramolecular Schmidt reaction to the asymmetric-synthesis of (−)-indolizidine-209b from pulegone. Heterocycles.

[95] Satake A., Shimizu I. (1993). A chiral synthesis of (8r,8as)-hexahydro-8-methyl-5(1h)-indolizinone. Tetrahedron: Asymmetry.

[96] Miyata O., Takahashi S., Tamura A., Ueda M., Naito T. (2008). Novel synthesis of substituted pyrrolidines and piperidines via radical addition—Ionic cyclization reaction of oxime ethers. Tetrahedron.

[97] Zhang L., Kim J.B., Jang D.O. (2014). Lewis acid-catalyzed cascade radical addition/cyclization for the synthesis of 3-substituted isoindolin-1-one derivatives. Tetrahedron Lett..

[98] Miyabe H. (2012). Inter- and intramolecular carbon-carbon bond-forming radical reactions. Synlett.

[99] Bertrand M.P., Feray L., Nouguier R., Stella L. (1998). 1,3-stereoinduction in radical additions to glyoxylate imines. Synlett.

[100] Kim B.H., Curran D.P. (1993). Asymmetric thermal-reactions with oppolzer camphor sultam. Tetrahedron.

[101] Miyabe H., Fujii K., Naito T. (1999). Highly diastereoselective radical addition to oxime ethers: Asymmetric synthesis of beta-amino acids. Org. Lett..

[102] Miyabe H., Fujii K., Naito T. (2003). Radical addition to oxime ethers for asymmetric synthesis of beta-amino acid derivatives. Org. Biomol. Chem..

[103] Friestad G.K., Qin J. (2000). Highly stereoselective intermolecular radical addition to aldehyde hydrazones from a chiral 3-amino-2-oxazolidinone. J. Am. Chem. Soc..

[104] Friestad G.K., Qin J. (2001). Intermolecular alkyl radical addition to chiral N-acylhydrazones mediated by manganese carbonyl. J. Am. Chem. Soc..

[105] Friestad G.K., Marie J.C., Deveau A.M. (2004). Stereoselective Mn-mediated coupling of functionalized iodides and hydrazones: A synthetic entry to the tubulysin gamma-amino acids. Org. Lett..

[106] Friestad G.K., Draghici C., Soukri M., Qin J. (2005). Radical addition approach to asymmetric amine synthesis: Design, implementation, and comparison of chiral N-acylhydrazones. J. Org. Chem..

[107] Friestad G.K., Marie J.C., Suh Y.S., Qin J. (2006). Mn-mediated coupling of alkyl iodides and chiral N-acylhydrazones: Optimization, scope, and evidence for a radical mechanism. J. Org. Chem..

[108] Korapala C.S., Qin J., Friestad G.K. (2007). Quinine synthesis studies: A radical-ionic annulation via Mn-mediated addition to chiral *N*-acylhydrazones. Org. Lett..

[109] Friestad G.K., Ji A. (2008). Mn-mediated coupling of alkyl iodides and ketimines: A radical addition route to α,α-disubstituted α-aminoesters. Org. Lett..

[110] Friestad G.K., Banerjee K. (2009). Synthesis of γ-amino esters via Mn-mediated radical addition to chiral γ-Hydrazonoesters. Org. Lett..

[111] Miyabe H., Ushiro C., Ueda M., Yamakawa K., Naito T. (2000). Asymmetric synthesis of alpha-amino acids based on carbon radical addition to glyoxylic oxime ether. J. Org. Chem..

[112] Friestad G.K., Shen Y.H., Ruggles E.L. (2003). Enantioselective radical addition to N-acyl hydrazones mediated by chiral Lewis acids. Angew. Chem. Int. Ed..

[113] Hu J., Wang J., Nguyen T.H., Zheng N. (2013). The chemistry of amine radical cations produced by visible light photoredox catalysis. Beilstein J. Org. Chem..

[114] Albini A., Mella M., Freccero M. (1994). A new method in radical chemistry—Generation of radicals by photoinduced electron-transfer and fragmentation of the radical-cation. Tetrahedron.

[115] Pandey G., Rani K.S., Lakshmaiah G. (1992). Direct carbon-carbon bond formation strategy at α-position of tertiary-amines by photoinduced electron-transfer (PET) processes. Tetrahedron Lett..

